# Predictors of acute intracranial hemorrhage and recurrence of chronic subdural hematoma following burr hole drainage

**DOI:** 10.1186/s12883-020-01669-5

**Published:** 2020-03-13

**Authors:** Fu Mei Chen, Ke Wang, Kang Li Xu, Li Wang, Tian Xiang Zhan, Fei Cheng, Hao Wang, Zuo-Bing Chen, Liang Gao, Xiao Feng Yang

**Affiliations:** 1 Emergency and Trauma Center, The International Medical Center, The First Affiliated Hospital, College of Medicine, Zhejiang University, 79 Qingchun Road, Hangzhou, 310003 Zhejiang Province China; 2 Department of Neurosurgery, Shanghai Tenth People’s Hospital, Tongji University, 301Yan Chang road, Shanghai, 200072 China; 3 Intensive Care Unit, Sir Run Run Shaw Hospital, College of Medicine, Zhejiang University, Hangzhou, 310016 Zhejiang Province China; 4 Department of Neurosurgery, The First Affiliated Hospital, College of Medicine, Zhejiang University, 79 Qingchun Road, Hangzhou, 310003 Zhejiang Province China; 5 Pathology department, The First Affiliated Hospital, College of Medicine, Zhejiang University, 79 Qingchun Road, Hangzhou, 310003 Zhejiang Province China; 6 Department of Rehabilitation Medicine, The First Affiliated Hospital, College of Medicine, Zhejiang University, 79 Qingchun Road, Hangzhou, 310003 Zhejiang Province China; 7 Emergency and Trauma Center, The International Medical Center, The First Affiliated Hospital, College of Medicine, Zhejiang University, 79 Qingchun Road, Hangzhou, 310003 Zhejiang Province China

**Keywords:** Chronic subdural hematoma, Acute intracranial hemorrhage, Recurrence, Burr hole drainage, Intraoperative irrigation

## Abstract

**Background:**

To investigate predictors of postoperative acute intracranial hemorrhage (AIH) and recurrence of chronic subdural hematoma (CSDH) after burr hole drainage.

**Methods:**

A multicenter retrospective study of patients who underwent burr hole drainage for CSDH between January 2013 and March 2019.

**Results:**

A total of 448 CSDH patients were enrolled in the study. CSDH recurrence occurred in 60 patients, with a recurrence rate of 13.4%. The mean time interval between initial burr hole drainage and recurrence was 40.8 ± 28.3 days. Postoperative AIH developed in 23 patients, with an incidence of 5.1%. The mean time interval between initial burr hole drainage and postoperative AIH was 4.7 ± 2.9 days. Bilateral hematoma, hyperdense hematoma and anticoagulant drug use were independent predictors of recurrence in the multiple logistic regression analyses. Preoperative headache was an independent risk factor of postoperative AIH in the multiple logistic regression analyses, however, intraoperative irrigation reduced the incidence of postoperative AIH.

**Conclusions:**

This study found that bilateral hematoma, hyperdense hematoma and anticoagulant drug use were independently associated with CSDH recurrence. Clinical presentation of headache was the strongest predictor of postoperative AIH, and intraoperative irrigation decreased the incidence of postoperative AIH.

## Introduction

Chronic subdural hematoma (CSDH) is one of the most common types of intracranial hemorrhage; however, its pathogenic mechanism remains unclear. The incidence of CSDH is ~ 3.4–5/100000 per year in the general population and 60–80/100000 per year in those aged ≥65 [1–5]. The treatment is often surgical evacuation, using techniques such as twist-drill craniotomy and burr hole drainage (BHD) or craniotomy. BHD is the most widely used technique, and has a satisfactory outcome, but has a CSDH recurrence rate of 4–38% [6–12]. Several studies have revealed that predictors of CSDH recurrence following surgical evacuation include age, sex, antiplatelet or anticoagulation therapy, Glasgow Coma Scale (GCS), diabetes mellitus, hypertension, bilateral hematoma, preoperative hematoma size, midline shift, hematoma density, intraoperative irrigation, type of surgery and postoperative air collection, however, these results are inconsistent [7, 13–19]. The more controversial risk factors include surgery type and intraoperative irrigation [9, 10, 20–22]. Previous reports have shown a CSDH mortality rate of 1.8–32% [6, 23].

Surgical complications, such as irritability, wound infection and acute intracranial hemorrhage (AIH), occur at a rate of 0–38% [24–26]. AIH, in particular, can cause severe neurological dysfunction, leading to a poor prognosis. However, the mechanism is still unclear and there is limited information on risk factors associated with AIH following surgery for CSDH. Some previous studies have evaluated recurrence risk following CSDH surgery using only a single or few predictors, however, the majority of these were single-center retrospective studies and lacked interaction between other variables and confounding factors. It is, therefore, important to identify the clinical and perioperative risk factors associated with AIH and CSDH recurrence following BHD surgery. In this multi-center retrospective study we evaluated the clinical factors associated with postoperative AIH and CSDH recurrence, to determine an optimal perioperative management strategy for BHD with or without irrigation in CSDH patients.

## Methods

We retrospectively analyzed the medical records of all patients diagnosed with CSDH at the Department of Neurosurgery, the First Affiliated Hospital, College of Medicine, Zhejiang University, Hangzhou, China between September 2015 and March 2019 (*n* = 315), and the Department of Neurosurgery of Shanghai Tenth People’s Hospital, Shanghai, China between January 2013 and May 2016 (*n* = 133). The patients were diagnosed by computed tomography (CT) or magnetic resonance imaging (MRI).

### Inclusion and exclusion criteria

The inclusion criteria were as follows: all patients were diagnosed with CSDH by CT or MRI; and initially underwent BHD with or without irrigation at one of the two institutions. Exclusion criteria included: CSDH that was treated conservatively or by surgical methods other than BHD (such twist-drill craniotomy and formal craniotomy with excision of subdural membrane); any other intracranial pathologies or intracranial surgery, such as ventriculoperitoneal shunt, epidural hematoma, parenchymal hemorrhage, intraventricular hemorrhage or ischemic insult; and recurrent CSDH.

### Definitions

In this study, we defined CSDH as subdural hematoma that could be treated with BHD. We defined postoperative recurrence of CSDH as a subsequent radiographic discharge following the primary treatment, with re-bleeding and/or increased size of the subdural hematomas on the operated side, with or without any clinical presentation. AIH was identified as a hyperdense lesion in the ventricle, brain parenchyma, subarachnoid, subdural space or epidural space, visible on CT scan during hospitalization, and accompanied by neurological deterioration. Chronic intracranial hemorrhage (CIH) in this study refers to cerebral hemorrhage other than recurrent CSDH or AIH.

The hematoma was classified as hypodense [< 30 Hounsfield units (HU)], isodense (30–60 HU), hyperdense (> 60 HU), or mixed density based on the density difference of the hematoma and the brain parenchyma. Patients with bilateral CSDH of different types were attributed according to which reflected the greater bleeding tendency.

Surgical evacuation of CSDH was carried out when the following indications were observed:

1. Unilateral or bilateral CSDH with maximum thickness > 10 mm and/or midline shift > 7 mm;
2. CSDH of any thickness causing mass effect, midline shift, or neurological signs and symptoms, such as focal deficit, and mental status changes;
3. Progressive increase in size of CSDH as observed through a series of CT or MRI scans.

In bilateral CSDH, the decision to evacuate one or both sides was generally made based on hematoma size and neurological symptoms. (i) Unilateral evacuation of bilateral CSDH: when the maximum diameter of the CSDH on the operation side was > 10 mm, when the contralateral hematoma was < 10 mm thick, and when the laterality of neurological symptoms could only be attributed to the thicker hematoma. (ii) Bilateral evacuation of bilateral CSDH: both sides received the same treatment in a single operation.

### Surgical procedures and general management

In all cases, a single burr hole was made under anesthesia. Local or general anesthetic was selected depending on the patient’s general status and ability to co-operate. A 4–5 cm vertical skin incision was made at the superior temporal line near the coronal suture or at the point of maximum thickness of the CSDH. A 1.5–2 cm craniotomy was performed. After opening the dura and outer membrane of the hematoma, a soft silicon tube was inserted into the subdural space through the burr hole overlying the large part of the subdural cavity, and tunneled for a minimum of 5 cm away from the scalp incision. Irrigation was not carried out in all surgeries. If carried out, irrigation was with 0.9% normal saline via the tube, until the output was clear. The drain was connected to a soft collection bag and the drainage tube was removed when there was no more drainage fluid. The patient stayed in bed until the drain was removed. If CT scan showed that subdural collections remained in the hematoma cavity after the operation, the patient was treated with urokinase, according to the surgeon’s preference. CT scanning indicated if the affected brain region showed sufficient re-expansion or when the amount decreased to < 50 ml per day.

All patients had their anticoagulant or antiplatelet status optimized prior to surgery, ensuring a preoperative international normalized ratio (INR) between 0.8 and 1.2. It was managed in various ways depending on the type of anticoagulant treatment. Agents used to reverse anticoagulant or antiplatelet medications were vitamin K1, thrombocytes, blood plasma, tranexamic acid, prothrombin complex concentrate, and desmopressin analogs. Antiplatelet or anticoagulant agents were usually re-started 1 month from the day of surgery.

Following CSDH diagnosis, some patients were given atorvastatin (20 mg per night) prior to the operation. The decision to give the patient preoperative atorvastatin was made by the neurosurgeon. In general, atorvastatin was discontinued for at least 1 month after surgery or based on follow-up imaging. All patients were followed-up for at least 12 months.

### Data collection

Demographic, clinical and radiographic data were collected using a standardized case report form and the information was added to a database. The following data were included: gender, age, history of anticoagulant or antiplatelet, head trauma event, GCS score on admission, medical history (e.g., hypertension, diabetes), preoperative main clinical symptoms (e.g., headache, dizziness), operation side (unilateral, unilateral evacuation of bilateral CSDH, bilateral evacuation of bilateral CSDH), hematoma density characteristics on CT (hyperdense, hypodense, mixed density, isodense), hematoma maximal thickness (preoperative hematoma thickness on the axial cut that showed the maximal diameter), midline shift, anesthesia method, intraoperative irrigation (non-irrigation versus irrigation), duration of drainage (days), urokinase injection after operation, complications (including acute intracranial bleeding, fever > 38 °C, seizure, irritability, poor wound healing and intracranial infection), time interval between initial operation and recurrence (days), time interval between initial operation and bleeding (days), and hospitalization time (days). All CT interpretations and measurements were made by a neurosurgeon and recorded after corroborating with the neuroradiologist’s report.

### Statistical analyses

SPSS software (version 16.0) was used for the statistical analysis.

Continuous data are expressed as the mean ± standard deviation (SD) and categorical data are expressed as the median value (IQR). Univariable (Student t-test and the Mann–Whitney U-test), bivariable (Chi-square) and multivariable (multiple logistic regression) analyses were performed to determine which, if any, of the studied variables were associated risk factors of CSDH recurrence and AIH. The variables with *P* < 0.1 in the univariate analysis were included in the multivariate model. Statistical significance was defined as *P*-value < 0.05.

## Results

### Baseline clinical characteristics of patients

A total of 448 patients (354 males, 94 females) were included in the analysis. The mean age was 68.1 ± 12.4 years (range: 14–98 years). CSDH recurrence occurred in 60 (13.4%) patients, including 26 with unilateral BDH evacuation, 11 with unilateral evacuation of bilateral CSDH, and 23 with bilateral evacuation of bilateral CSDH. Complications occurred in 122 patients (27.2%), including 23 (5.1%) with AIH (16 patients with unilateral evacuation, five with unilateral evacuation of bilateral CSDH, and two with bilateral evacuation of bilateral CSDH). One patient died (mortality rate = 0.2%). The mean duration of hospitalization time was 14.0 ± 5.5 days (range: 3–42 days). Patient information is provided in Table 1.

**Table 1:** Clinical characteristics of patients

Variables	No. (%)
Total patients	448
Gender	
Male	354 (79.0)
Female	94 (21.0)
Age, mean ± SD, years	68.1 ± 12.4 (14.0–98.0)
Head trauma event	295 (65.8)
Medical history	
Hypertension	172 (38.4)
Diabetes	59 (13.2)
Heart disease	47 (10.5)
Cerebral infarction	28 (6.3)
Renal failure dialysis	3 (0.7)
Antiplatelet or anticoagulant use	
Antiplatelet use	32 (7.1)
Anticoagulant use	15 (3.3)
Preoperative clinical symptoms	
Headache	202 (45.1)
Dizziness	139 (31.0)
Motor weakness	253 (56.5)
Nausea/vomiting	31 (6.9)
Speech impairment	17 (3.8)
Unsteady gait	8 (1.8)
Incontinence	11 (2.5)
Memory impairment	14 (3.1)
GCS at admission	
15	385 (85.9)
3–14	63 (14.1)
Operation side	
Unilateral hematoma	294 (65.6)
Bilateral hematoma	154 (34.4)
Bilateral evacuated unilaterally	54 (12.1)
Bilateral evacuated bilaterally	100 (22.3)
Radiological characteristics	
Midline shift	
< 10 mm	292 (65.2)
≥ 10 mm	156 (34.8)
Preoperative maximal thickness	
< 20 mm	99 (22.1)
≥ 20 mm	349 (77.9)
Hematoma density	
Hyperdense	133 (29.7)
Hypodense	71 (15.8)
Mixed density	210 (46.9)
Iso-dense	34 (7.6)
Treatment	
Atorvastatin perioperatively	171 (38.2)
Anesthesia	
Local	201 (44.9)
General	247 (55.1)
Intraoperative irrigation	
Irrigation	376 (83.9)
Non-irrigation	72 (16.1)
Injection urokinase after operation	12 (2.7)
Duration of drainage, day	
1	63 (14.1)
2	306 (68.3)
≥ 3	79 (17.6)
Outcomes and complications	
Clinical symptoms changes at discharge	
Improvement	384 (85.7)
Unchanged	19 (4.3)
Deterioration	45 (10.0)
GCS at discharge	
Improvement	62 (13.8)
Unchanged	373 (83.3)
Deterioration	13 (2.9)
Complications	122 (27.2)
Fever	69 (15.4)
Irritability	19 (4.3)
Poor wound healing	1 (0.2)
Seizure	2 (0.4)
Deep vein thrombosis	1 (0.2)
AIH	23 (5.1)
CIH	7 (1.6)
Recurrence	60 (13.4)
Mortality	1 (0.2)
Hospitalization time, mean ± SD, days	14.0 ± 5.5 (3.0–42.0)

*GCS* Glasgow Coma Scale, *AIH* Acute intracranial hemorrhage, *CIH* Chronic intracranial hemorrhage

### Analysis of patients with recurrence

Sixty patients (53 males, seven females) experienced CSDH recurrence following the initial BHD. The mean age was 69.6 ± 10.8 years (range: 31–88 years). The mean time interval between initial BHD and recurrence was 40.8 ± 28.3 days (range: 12–180 days).

Univariate analysis (Table 2) showed that risk factors including anticoagulant drugs (*P* = 0.038), density of hematoma (*P* = 0.009), operation side (*P* < 0.001), and atorvastatin administration (*P* = 0.021) were significantly related to CSDH recurrence. Unilateral or bilateral evacuation of bilateral CSDH were not significantly correlated with CSDH recurrence following BHD (2.5% versus 5.1%, *P* = 0.707). Although there was no significant correlation between gender and recurrence (*P* < 0.1), it was included in the multivariate analysis according to our multivariate inclusion analysis criteria. Other variables that were not significantly correlated with CSDH recurrence are shown in Table 2.

**Table 2:** Univariate analysis of factors for recurrence of CSDH

Variables	Recurrence	No recurrence	*P* Value
Patients	60(%)	388(%)	
Gender			0.057
Male	53 (88.3)	301 (77.6)	
Female	7 (11.7)	87 (22.4)	
Age (years)			0.576
< 70	30 (50)	209 (53.9)	
≥ 70	30 (50)	179 (46.1)	
Head trauma event	35 (58.3)	260 (67.0)	0.187
Medical history			
Hypertension	25 (41.7)	147 (37.9)	0.575
Diabetes	10 (16.7)	49 (12.6)	0.389
Heart disease	10 (16.7)	37 (9.5)	0.093
Cerebral infarction	5 (8.3)	23 (5.9)	0.404
Renal failure dialysis	1 (1.7)	2 (0.5)	0.351
Antiplatelet or anticoagulant drugs			
Antiplatelet drugs	6 (10.0)	26 (6.7)	0.415
Anticoagulant drugs	5 (8.3)	10 (2.6)	0.038
The main symptoms			
Headache	26 (43.3)	176 (45.4)	0.769
Dizziness	17 (28.3)	122 (31.4)	0.628
Motor weakness	32 (53.3)	221 (57.0)	0.598
Nausea/vomiting	2 (3.3)	29 (7.5)	0.408
Speech impairment	3 (5.0)	14 (3.6)	0.486
Unsteady gait	2 (3.3)	6 (1.5)	0.292
Incontinence	1 (1.7)	10 (2.6)	1.000
Memory decline	1 (1.7)	13 (3.4)	0.704
GCS at admission			0.566
15	53 (88.3)	332 (85.6)	
3–14	7 (11.7)	56 (14.4)	
Operation side			*P* < 0.001
Unilateral hematoma	26 (43.3)	270 (69.6)	
Bilateral hematoma	34 (56.7)	118 (30.4)	
Radiological characteristics			
Midline shift			0.975
< 10 mm	39 (65.0)	253 (65.2)	
≥ 10 mm	21 (35.0)	135 (34.8)	
Preoperative maximal thickness			0.154
< 20 mm	9 (15.0)	90 (23.2)	
≥ 20 mm	51 (85.0)	298 (76.8)	
Hematoma density			0.009
Hyper-dense	29 (48.3)	104 (26.8)	
Hypo-dense	7 (11.7)	64 (16.5)	
Mixed density	21 (35.0)	189 (48.7)	
Iso-dense	3 (5.0)	31 (8.0)	
Treatment			
Atorvastatin perioperatively	31 (51.7)	140 (36.1)	0.021
Anesthesia			0.982
Local	27 (45.0)	174 (44.8)	
General	33 (55.0)	214 (55.2)	
Intraoperative irrigation	49 (81.7)	327 (84.3)	0.608
Injection urokinase after operation	3 (5.0)	9 (2.3)	0.208
Duration of drainage, day			0.842
1	9 (15.0)	54 (14.0)	
2	42 (70.0)	264 (68.0)	
≥ 3	9 (15.0)	70 (18.0)	
Complications			
Fever	5 (8.3)	64 (16.5)	0.103
Irritability	4 (6.7)	15 (3.9)	0.303
Poor wound healing	1 (1.7)	0	–
Seizure	1 (1.7)	1 (0.26)	0.250
Deep vein thrombosis	0	1 (0.26)	–
AIH	0	23 (5.9)	–
Hospitalization time, mean ± SD, days	14.0 ± 6.4	14.0 ± 5.4	0.905

*GCS* Glasgow Coma Scale, *AIH* Acute intracranial hemorrhage

In the multivariate logistic regression model, bilateral hematoma [Odds ratio (OR), 2.563; 95% confidence interval (CI), 1.439–4.563; *P* = 0.001], mixed density (OR, 0.433; 95%CI, 0.229–0.818; *P* = 0.010, hyperdense as reference) and anticoagulant drugs (OR, 4.309; 95%CI, 1.244–14.923; *P* = 0.021) were significantly associated with CSDH recurrence. Other variables not significantly associated with CSDH recurrence are detailed in Table 3.

**Table 3:** Multivariate logistic regression analysis of factors for recurrence of CSDH

Risk factors	OR	95% CI	***P*** value
Gender	2.139	0.897–5.099	0.086
Operation side (Unilateral vs Bilateral)	2.563	1.439–4.563	0.001
Hematoma density			
Hyperdense	Reference	Reference	Reference
Hypodense	0.428	0.173–1.061	0.067
Mixed density	0.433	0.229–0.818	0.010
Iso-dense	0.364	0.101–1.311	0.122
Atorvastatin perioperatively	1.634	0.918–2.908	0.095
Anticoagulant drugs	4.309	1.244–14.923	0.021

*OR* Odds ratio, *CI* Confidence interval

### Analysis of patients with acute intracranial hemorrhage

Twenty-three patients (15 males, eight females) experienced postoperative AIH. The mean age was 68.5 ± 11.5 years (range: 42–87 years). The mean time interval between initial BHD and postoperative AIH was 4.7 ± 2.9 days (range: 1–10 days).

Univariate analysis indicated that preoperative headache (*P* = 0.046), preoperative hematoma thickness (*P* = 0.043) and intraoperative irrigation (*P* = 0.020) were significantly related to postoperative AIH (Table 4). Operation side (*P* = 0.683) was not significantly related to postoperative AIH, and there was no significant difference in the incidence of postoperative AIH between unilateral or bilateral evacuation of bilateral CSDH (1.1% versus 0.4%, *P* = 0.052). Other variables with *P* > 0.1 in the univariate analysis are shown in Table 4.

**Table 4:** Univariate analysis of factors for postoperative AIH

Variables	AIH	No AIH	*P* Value
Patients	23(%)	425(%)	
Gender			0.113
Male	15 (65.2)	339 (79.8)	
Female	8 (34.8)	86 (20.2)	
Age (years)			0.908
< 70	12 (52.2)	227 (53.4)	
≥ 70	11 (47.8)	198 (46.6)	
Head trauma event	14 (60.9)	281 (66.1)	0.605
Medical history			
Hypertension	12 (52.2)	160 (37.6)	0.163
Diabetes	2 (8.7)	57 (13.4)	0.754
Heart disease	1 (4.3)	46 (10.8)	0.494
Cerebral infarction	1 (4.3)	27 (6.4)	1.00
Renal failure dialysis	0 (0)	3 (0.7)	–
Antiplatelet or anticoagulant drugs			
Antiplatelet drugs	3 (13.0)	29 (6.8)	0.221
Anticoagulant drugs	1 (4.3)	14 (3.3)	0.552
Preoperative clinical symptoms			
Headache	15 (65.2)	187 (44.0)	0.046
Dizziness	0 (0)	139 (32.7)	–
Motor weakness	14 (60.9)	239 (56.2)	0.662
Nausea/vomiting	2 (8.7)	29 (6.9)	0.668
Speech impairment	0 (0)	17 (4.0)	–
Unsteady gait	1 (4.3)	7 (1.6)	0.346
Incontinence	0 (0)	11 (2.6)	–
Memory decline	0 (0)	14 (3.3)	–
GCS at admission			0.756
15	21 (91.3)	364 (85.6)	
3–14	2 (8.7)	61 (14.4)	
Operation side			0.683
Unilateral hematoma	16 (69.6)	278 (65.4)	
Bilateral hematoma	7 (30.4)	147 (34.6)	
Radiological characteristics			
Midline shift			0.367
< 10 mm	17 (73.9)	275 (64.7)	
≥ 10 mm	6 (26.1)	150 (35.3)	
Preoperative maximal thickness			0.043
< 20 mm	9 (39.1)	90 (21.2)	
≥ 20 mm	14 (60.9)	335 (78.8)	
Hematoma density			0.924
Hyper-dense	7 (30.4)	126 (29.6)	
Hypo-dense	4 (17.4)	67 (15.8)	
Mixed density	10 (43.5)	200 (47.1)	
Iso-dense	2 (8.7)	32 (7.5)	
Treatment			
Atorvastatin perioperatively	9 (39.1)	162 (38.1)	0.922
Anesthesia			0.249
Local	13 (56.5)	188 (44.2)	
General	10 (43.5)	237 (55.8)	
Intraoperative irrigation	15 (65.2)	361 (84.9)	0.020
Injection urokinase after operation	0 (0)	12 (2.8)	–
Duration of drainage, day			0.307
1	1 (4.3)	62 (14.6)	
2	19 (82.7)	287 (67.5)	
≥ 3	3 (13.0)	76 (17.9)	

*GCS* Glasgow Coma Scale, *AIH* Acute intracranial hemorrhage

Multivariate analysis showed that preoperative headache (OR, 3.053; 95%CI, 1.181–7.890; *P* = 0.021) and intraoperative irrigation (OR, 0.289; 95%CI, 0.114–0.735; *P* = 0.009) were significantly associated with postoperative AIH. Other variables not significantly associated with postoperative AIH or CSDH recurrence are shown in Table 5.

**Table 5:** Multivariate logistic regression analysis of factors related to postoperative AIH

Risk factors	OR	95% CI	***P*** value
Headache	3.053	1.181–7.890	0.021
Preoperative maximal thickness	0.543	0.219–1.351	0.189
Intraoperative irrigation	0.289	0.114–0.735	0.009

*OR* Odds ratio, *CI* Confidence interval

## Discussion

CSDH is one of the most commonly treated neurosurgical disorders and BHD is a universal surgical treatment [11, 18, 27]. The rate of CSDH recurrence following BHD is 4–38% and the complications rate is 0–38% [6–12, 24, 26]. In this study, we found the CSDH recurrence rate after BDH was 13.4% and the complication rate was 27.2%. Of the 122 patients with complications, 23 had postoperative AIH (5.1% incidence). Pang et al. similarly reported an incidence of postoperative AIH of 4.57% [25]. According to the literature, the mortality rate due to CSDH is 1.8–32%, however, in this study, only one patient died of respiratory failure, giving a lower mortality rate of 0.2% [6, 23]. Comparing our results with previous studies, our definition of CSDH, postoperative recurrence of CSDH and postoperative AIH are in accordance with medical norms and logic.

CSDHs are usually unilateral, but do present as bilateral in ~ 9.2–34.9% of cases [5, 28–30]. However, not all patients with bilateral CSDH warrant evacuation of both sides at initial presentation. The major determinants of unilateral or bilateral evacuation are preoperative maximal thickness, the degree of midline shift, and lateralized clinical symptoms, followed by surgical treatment of only the largest or the symptomatic hematoma, leaving the other hematoma non-operated [31]. In our study, 154 patients (34.4%) had bilateral CSDH. Fifty-four (12.1%) had unilateral evacuation and 100 (22.3%) were evacuated bilaterally. Previous studies have shown that simultaneous bilateral decompression in bilateral CSDH reduces complications and the rate of CSDH recurrence, however, others have not corroborated this result [31–33]. In our study, there was no significant difference between the rate of CSDH recurrence and incidence of AIH following unilateral or bilateral evacuation in bilateral CSDH.

The recurrence rate in unilateral CSDH is lower than that of bilateral CSDH, making bilateral hematoma a risk factor for CSDH recurrence [16, 19]. This could be due to poor brain re-expansion in bilateral CSDH compared to unilateral CSDH, which may result in a brain parenchymal shift, tearing of the blood vessels, postoperative pneumocephalus and cerebrospinal fluid accumulation in the hematoma cavity, leading to a higher recurrence rate [19, 34, 35]. Another theory is that patients with bilateral CSDH tend to have a history of brain atrophy, which leads to poor re-expansion, resulting in a higher recurrence rate [19, 35]. In our study, bilateral hematoma was found to be a risk factor for postoperative recurrence of CSDH, but was not a risk factor for postoperative AIH. Whether the brain re-expansion theory or the brain atrophy theory were correct will require time to accumulate results. In our study, the mean time interval (4.7 ± 2.9 days) between initial surgery and postoperative AIH was significantly lower than the mean time interval (40.8 ± 28.3 days) between initial surgery and postoperative recurrence.

Some studies have reported that male sex is an independent risk factor of CSDH recurrence, and that males likely have more trauma and more frequent complications than females. However, other studies have suggested that gender is not associated with CSDH recurrence [13, 36]. In our study, 295 patients had a history of trauma, including 233 males and 62 females. According to multivariate logistic regression analysis, there was no significant correlation between gender and CSDH recurrence. To date, no literature has reported the relationship between gender and postoperative AIH. We found that gender was not significantly associated with postoperative AIH.

The clinical presentation of CSDH varies from asymptomatic to headache, limb weakness, incontinence, etc. According to our study, preoperative headache is an independent risk factor for postoperative AIH. When CSDH presents as a clinical manifestation of headache, it indicates a higher intracranial pressure and a secondary cerebral blood flow reduction in the deep cerebral regions, causing blood vessels to spasm and increasing the risk of vascular accidents [37]. Thus, when blood flow in the brain changes or intracranial pressure changes significantly in a short period of time, blood vessels rupture, resulting in bleeding.

Many studies suggest that preoperative hematoma density is associated with CSDH recurrence, and hyperdense hematoma is an independent risk factor for CSDH recurrence [13, 38]. In present study, although nearly 1/3 of the patients had hyperdense imaging findings, according to our clinical experience and our definition of CSDH, neurosurgeons evaluated patients with subdural hematoma no matter what the density of subdural hematoma on imaging, once they were able to be treated with BHD, these patients could be managed as patients with CSDH. In our study, hyperdense hematoma were more prone to CSDH recurrence than mixed density (OR, 0.433; 95%CI, 0.229–0.818; *P* = 0.010, hyperdense as reference). The relationship between hematoma density and postoperative AIH has not been reported in the literature. In our study, hematoma density was not significantly associated with postoperative AIH. We suspect that when hyperdense hematoma are compared with the other three types of hematoma density, repeated microhemorrhages from the immature capillary network in the outer membrane take place and accumulate over time, causing CSDH recurrence [38].

Preoperative hematoma thickness has been reported as an independent risk factor for CSDH recurrence [7]. It is thought that hematoma thickness has an increased tendency to recur because the subdural space is larger than a small lesion postoperatively [7, 36]. As hematoma thickness is related to many factors, such as age, midline shift and brain atrophy, it may not be a useful predictor of CSDH recurrence and postoperative AIH. However, other studies have shown no significant relationship between hematoma thickness and CSDH recurrence [13, 39], and the relationship between hematoma thickness and postoperative AIH has not been reported. In our study, the hematoma thickness of 20 mm was selected as a threshold, compared with the trauma subdural hematoma guidelines threshold of 10 mm, there were two reasons. First, a part of the patients were non-traumatic chronic subdural hematomas; second, according to our clinical experience, when the thickness of hematoma is between 10 mm and 20 mm, some patients had no obvious clinical manifestations and did not require surgical treatment. According to the results, there was no significant relationship between hematoma thickness and CSDH recurrence or postoperative AIH.

Our results showed that anticoagulant drug use is an independent risk factor for CSDH recurrence. Antiplatelet drugs did not increase the CSDH recurrence rate following BHD. Similarly, a recent systematic study showed that anticoagulant drugs increased re-bleeding on the operation side of CSDH, but antiplatelet therapy did not [40]. However, this result is controversial as in other studies, antiplatelets or anticoagulants were not associated with CSDH recurrence [16, 36, 41]. The authors speculated that the reason for this finding may be that the patients were not adequately anticoagulated, especially in the non-recurrence group. At present, only one study has reported that anticoagulants or antiplatelet drugs do not increase the incidence of postoperative AIH [25]. Similar to our results, we suspect that it may be the result offset caused by insufficient sample size. Therefore, a large sample clinical trial is necessary to verify this result.

Atorvastatin is another management option, which may be used alone or in conjunction with surgery. Several studies have shown that CSDH is caused by impaired angiogenesis in the neomembrane and localized inflammation, and that atorvastatin improves angiogenesis and reduces the inflammatory response [42, 43]. A randomized control study showed that the oral administration of atorvastatin is safe and effective in treating CSDH, and promotes the resolution of hematoma [44]. A prospective study showed that atorvastatin administration may decrease the risks of CSDH recurrence [45]. However, in our study, atorvastatin did not reduce the rate of CSDH recurrence, nor did it reduce the incidence of postoperative AIH. We suspect that different research methods may lead to varying results.

In our study, intraoperative saline irrigation was based on the surgeon’s preference. Some studies have shown that intraoperative saline irrigation of the hematoma cavity does not improve the incidence of postoperative AIH and CSDH recurrence [25, 46]. According to our results, intraoperative irrigation does not reduce the risk of CSDH recurrence, however, it does reduce the incidence of postoperative AIH. We speculate that BHD for CSDH without irrigation significantly reduces intracranial pressure for a short period of time. The abrupt decrease in intracranial pressure may cause further damage to the intracranial blood vessels and lead to acute intracranial bleeding.

Our study had some limitations. First, our study is a retrospective design, which usually produces a risk of bias. Based on our data and personal communication with neurosurgeons in each participating department, neurosurgeons not only choose the surgical method based on symptoms and hematoma size, but also according to their preferences. For example, the use of a single burr hole, the 1.5-2 cm craniotomy and whether to irrigate is based on neurosurgeon preference. However, according to literature reports and our results, the selection of these surgical methods did not result in significant differences postoperative complications in total and recurrence rates [25]. Second, in the multivariate model, although ideally more stringent criteria for variable inclusion in the model would be applied in lieu of *p* < 0.1. However, it may lead to too few variables and too many confounding factors, which will have a greater impact on the results.

## Conclusions

In this study, we evaluated predictors of CSDH recurrence and postoperative AIH following BHD in a multi-center retrospective study of patients with CSDH. We identified bilateral hematoma, hyperdense hematoma and anticoagulant drugs as independent risk factors for recurrence of CSDH. Preoperative headache is an independent risk factor for postoperative AIH, and intraoperative irrigation reduces the incidence of postoperative AIH. Further prospective multi-center studies will be required to evaluate these findings.

## Data Availability

The datasets used during the current study are available from the corresponding author on reasonable request.

## Abbreviations

AIH: Acute intracranial hemorrhage
BHD: Burr hole drainage
CIH: Chronic intracranial hemorrhage
CSDH: Chronic subdural hematoma
CT: Computed tomography
GCS: Glasgow coma scale
MRI: Magnetic resonance imaging
